# Nicotinic acid protects germinal vesicle oocyte meiosis against toxicity of benzo(a)pyrene in mice and humans

**DOI:** 10.1530/REP-24-0364

**Published:** 2025-03-19

**Authors:** Min Gao, Yanling Qiu, Dungao Li, Shaoquan Zhan, Bohong Chen, Tianqi Cao, Junjiu Huang, Zhiyun Chen

**Affiliations:** ^1^ MOE Key Laboratory of Gene Function and Regulation, State Key Laboratory of Biocontrol, School of Life Sciences, Sun Yat-sen University, Guangzhou, China; ^2^ Key Laboratory of Reproductive Medicine of Guangdong Province, School of Life Sciences and the First Affiliated Hospital, Sun Yat-sen University, Guangzhou, China; ^3^ Hainan Provincial Key Laboratory for Human Reproductive Medicine and Genetic Research, Hainan Provincial Clinical Research Center for Thalassemia, Reproductive Medical Center, International Technology Cooperation Base ‘China-Myanmar Joint Research Center for Prevention and Treatment of Regional Major Disease’ By the Ministry of Science and Technology of China, The First Affiliated Hospital of Hainan Medical University, Hainan Medical University, Haikou, China; ^4^ Key Laboratory of Reproductive Health Diseases Research and Translation of Ministry of Education, The First Affiliated Hospital, Hainan Medical University, Haikou, China; ^5^ The Reproduction Medicine Center of Huizhou Central People's Hospital, Huizhou, China; ^6^ Key Laboratory of Reproductive Medicine of Guangdong Province, Third Affiliated Hospital of Guangzhou Medical University, Guangzhou, China

**Keywords:** benzo(a)pyrene, germinal vesicle oocyte, nicotinic acid, oxidative stress, *in vitro* maturation

## Abstract

**In brief:**

Low concentrations of benzo(a)pyrene in the follicular fluid of smokers disrupt oocyte maturation, leading to meiotic defects. Nicotinic acid (NA) partially rescues these defects, offering insights into potential strategies for protecting fertility.

**Abstract:**

Benzo(a)pyrene (BaP), a carcinogen present in cigarette smoke, was detected in human follicular fluid at concentrations of approximately 5 nM in smokers and 7 nM in cases of assisted reproductive failure. However, whether a low concentration of BaP affects germinal vesicle (GV) oocyte maturation remains unclear. Here, we investigated the effects of 5 nM BaP on GV oocyte maturation in both mice and humans. In mice, GV oocytes were treated with 5 or 50 nM BaP, while human oocytes were exposed to 5 nM BaP. Our results demonstrated that 5 or 50 nM BaP exposure significantly inhibited first polar body extrusion during oocyte maturation. Mechanistic investigations revealed that BaP treatment downregulated Sirt1 protein expression in both GV and metaphase II (MII) stage mouse oocytes. Moreover, BaP exposure induced multiple cellular abnormalities, including spindle disorganization, cortical actin cap disruption, mitochondrial dysfunction and DNA damage in MII oocytes. Importantly, 15 μM NA supplementation increased Sirt1 expression and significantly rescued most of the abnormal effects. Subsequently, 5 nM BaP exposure impaired meiotic progression by reducing mitochondrial membrane potential and causing significant reactive oxygen species accumulation in human GV oocytes. Importantly, 15 μM NA supplementation partially rescued human GV oocytes from the toxicity of BaP during *in vitro* maturation (IVM). The present study indicated that a low BaP concentration in follicular fluid can significantly disrupt GV oocyte IVM, inducing meiotic defects in both mice and humans. NA has been shown to provide partial protection to GV oocyte meiosis against the toxicity of BaP during IVM.

## Introduction

Smoking is a global health concern; approximately 177 million women worldwide are smokers (Singer *et al.* 2011). Tobacco smoking has been identified as a reproductive toxicant, with adverse effects including but not limited to ovarian failure and infertility (Neal *et al.* 2005, Waylen *et al.* 2009, Radin *et al.* 2014). It has been demonstrated that individuals who are nonsmokers yet are exposed to tobacco smoke for a considerable duration may experience reproductive consequences that are comparable to those observed in smokers (Practice Committee of the American Society for Reproductive Medicine 2018). More than 4,000 chemicals are present in cigarette smoke, and several of them exhibit toxic, mutagenic and carcinogenic properties. Benzo(a)pyrene (BaP) is a representative pollutant and carcinogen (Budani & Tiboni 2017). The main routes of BaP entry into the human body are tobacco smoke, coal tar and grilled foodstuffs.

Recently, the considerable toxicity of BaP to the female reproductive system has been the focus of mounting attention. BaP and its metabolites have been detected in the placenta, serum of pregnant women and breast milk produced by women who are in the lactation period (Madhavan & Naidu 1995, Al-Saleh *et al.* 2013). The pregnant women were exposed to BaP (2.27 mg/m^3^) in the air, which resulted in an increase in DNA adducts in neonatal blood. The DNA adducts were formed by epoxide metabolites (BPDE) of BaP, which were covalently bound to DNA. This increased the carcinogenic, teratogenic or mutagenic potential of the adducts (Sadeu & Foster 2011). Furthermore, elevated levels of reactive oxygen species (ROS) cause redox imbalance and mitochondrial dysfunction, which in turn have been shown to result in DNA damage, apoptosis and cellular aging (Sobinoff *et al.* 2012). Women exposed to mainstream smoke had significantly elevated levels of BaP (1.32 ± 0.68 ng/mL, about 5 nM) in follicular fluid compared to nonsmoking counterparts (0.03 ± 0.01 ng/mL) (Neal *et al.* 2008). The follicular fluid microenvironment can affect the quality of oocytes and even accelerate oocyte aging or lead to infertility (Ahmed *et al.* 2020). Immature oocytes were present in follicular fluid before ovulation. It is important to study the impact of BaP in follicular fluid on the maturation of germinal vesicle (GV) oocytes in exposed populations. However, the effect of the follicular fluid concentration of BaP on GV oocyte maturation remains unclear. Furthermore, it is important for the development of assisted reproductive techniques to determine whether small molecules can be found to rescue the toxicity of the substance.

Antioxidant strategies represent the primary method employed in addressing ovum hypo-evolutions caused by environmental pollutants. For instance, N-acetyl-L-cysteine (NAC) reduced ROS generated during *in vitro* maturation (IVM). The observed effect was found to be diminished upon the inhibition of Silencing Information Regulator 2 Related Enzyme 1 (SIRT1), thus indicating that SIRT1 plays a pivotal role in the mitigation of alterations in redox states during IVM (Di Emidio *et al.* 2014). In fact, the majority of the antioxidants used during IVM (such as melatonin and resveratrol) focused on detecting the protein levels of nicotinamide adenine dinucleotide (NAD^+^)-dependent deacetylase SIRT1 (Tamura *et al.* 1998, Lan *et al.* 2018, Nishigaki *et al.* 2022). NAD^+^ is a crucial coenzyme that plays a pivotal role in numerous significant biological processes and serves as a substrate for SIRT1. NAD^+^ not only accelerates the consumption of free radicals but also inhibits their production, which in turn maintains cellular free radical homeostasis. Loss of NAD^+^ alters the NAD^+^/SIRT1 axis and disrupts mitochondrial homeostasis (Croteau *et al.* 2017). NAD^+^ supplementation has been adopted for improving oocyte quality by reducing oxidative stress damage. NA is classified as a form of vitamin B3 and has been identified as a significant precursor of NAD^+^ (Wu *et al.* 2019, Lundt & Ding 2021). Collins *et al.* reported that NA is a more favorable precursor substance than NAM in the liver, intestine and kidneys. It has been demonstrated that the administration of nicotinamide adenine dinucleotide (NA) has the capacity to enhance the bioavailability of NAD^+^ precursors in the follicular fluid of dominant follicles in aging oocytes, thereby improving oocyte quality (Wu *et al.* 2019). Given the greater efficacy of NA *in vitro* supplementation, we elected to supplement NAD^+^ by adding NA and evaluated the role of NA by detecting Sirt1 protein levels.

Herein, we focused on the effects of 5 or 50 nM BaP on GV oocyte maturation in mice and humans. The results demonstrate that BaP exposure disrupts redox homeostasis, leading to adverse effects on maturation and quality in both mouse and human GV-MII oocyte development. NA supplementation significantly improved the quality of GV oocytes during IVM.

## Materials and methods

All chemicals and culture media were purchased from Sigma (USA) unless stated otherwise.

### Animal Care and ethics statement

CD1 mice were used in this study. They were housed in a specific pathogen - free (SPF) facility at 22 (± 2)°C and 50 (± 10)% humidity. Standard rodent chow and filtered, autoclaved water were provided *ad* *libitum*. A 12 - hour light/darkness cycle (lights on 8:00 am – 8:00 pm) was maintained. The protocols were approved by the Animal Care and Use Committee of Guangdong Medical University and were performed according to the Institutional Animal Care and Use Committee of Guangdong Medical University, People’s Republic of China. The approval number was GDY2202447. Female CD1 mice, 6–8 weeks old, were used in this study.

### Ethics statement on discarded human oocytes

The study in humans was approved by the Committee of Medical Ethics, and the approval number was ky112022040. The research has been carried out in accordance with the World Medical Association Declaration of Helsinki, and all patients provided written informed consent. GV oocyte collection was conducted at the Hospital of Huizhou Reproductive Medicine Center. The inclusion criteria of oocytes were defined as follows: intracytoplasmic sperm injection (ICSI) scheduled for the current cycle; age under 35 years; exclusion of infertility attributable to female factors; and a body mass index between 18.5 and 23.9 kg/m^2^.

### Mouse GV oocytes collection and culture

CD1 mice were used for oocyte collection. To collect fully grown GV oocytes, mice were superovulated with 5 IU of pregnant mare serum gonadotropin via intraperitoneal injection. For IVM, oocytes were cultured in M2 medium for approximately 14 h under liquid paraffin oil at 37°C in a 5% CO_2_ atmosphere.

### Human GV oocytes collection and culture

In humans, GV oocytes are not used for clinical purposes and are typically discarded in accordance with the policies of *in vitro* fertilization (IVF) programs. Discarded immature oocytes were utilized for research purposes only if patients had provided informed consent for the use of their discarded materials. The study utilized immature oocytes that were aspirated during oocyte retrieval from patients undergoing intracytoplasmic sperm injection in an IVF program between May 2019 and May 2022. In total, 78 human oocytes were collected over a 2-year period from individuals under 35 years of age. Human GV oocytes were frozen (VT101) and unfrozen (VT102) following the manufacturer’s instructions. The freezing process was conducted by doctors at the Reproduction Medicine Center of Huizhou Municipal Central Hospital, stored in a liquid nitrogen tank, and transported to the laboratory of the School of Life Sciences, Sun Yat-sen University. Before the experiment, the immature oocytes underwent an unfreezing process. Subsequently, they were cultured *in vitro* in G-IVF PLUS medium for 24–30 h under liquid paraffin oil at 37°C in a 5% CO_2_ atmosphere.

### BaP and NA treatment

BaP powder was purchased from Sigma (51968) and prepared as a 5 mM stock solubilized in dimethyl sulfoxide (DMSO). The stock was stored at −20°C and freshly diluted appropriately in culture medium to obtain a final concentration with an equivalent vehicle, DMSO (0.1%) as a negative control. Women exposed to mainstream smoke have approximately 5 nM BaP in their follicular fluid (Neal *et al.* 2008). Thus, 5 nM and a relatively high concentration of 50 nM were used. NA powder was purchased from Sigma (N0761) and added to the M2 maturation medium for *in vitro* supplementation. According to a previously reported concentration of oocytes treated with NA of 25–50 μM (Wang *et al.* 2021), we conducted NA concentration gradient screening to a final concentration of 7.5, 15 and 60 μM.

In the mouse studies, BaP dissolved in DMSO was diluted in M2 medium to produce final concentrations of 5 and 50 nM. The negative control group was treated with the same amount of DMSO (CTRL group). NA was dissolved in 5 or 50 nM BaP-contaminated M2 medium to yield final concentrations of 7.5, 15 and 60 μM. In humans, we explored the toxic effects of 5 nM BaP on GV oocyte maturation. BaP dissolved in DMSO was diluted in the G-IVF PLUS medium to produce a final concentration of 5 nM. The control group was treated with the same amount of DMSO (CTRL group). NA was dissolved in 5 nM BaP-contaminated culture medium to obtain a final concentration of 15 μM. A 2.5 μM concentration of milrinone (Sigma-Aldrich 1443908) was used to stop GV breakdown (GVBD) when collecting GV oocytes.

### Antibodies

Rabbit polyclonal anti-SIRT1 antibody was purchased from Abcam (Cat#: ab32441, UK). Rabbit polyclonal anti-γ-H2A.X (phosphor-S139) antibody was purchased from Abcam (Cat#: ab2893, UK).

### Plasmid construction and mRNA synthesis

Total RNA was extracted from mouse GV oocytes using the Arcturus PicoPure RNA Isolation Kit (Applied Biosystems, USA), and cDNA synthesis was performed using the QIAquick PCR Purification Kit (Qiagen, Germany). RT-PCR products were purified, digested with FseI and AscI (New England Biolabs, USA), and then cloned into the pCS2+ vector. To synthesize *Sirt1 *mRNA, the *Sirt1*-pCS2+ plasmids were linearized using NotI, and capped cRNAs were produced through *in vitro* transcription with SP6 mMessage mMachine (Thermo Fisher Scientific, USA) following the manufacturer’s instructions. The resulting mRNA was then stored at −80°C.

### Sirt1 overexpression

Microinjections of *Sirt1* mRNA were performed using a microinjector to overexpress Sirt1 in GV oocytes. In the overexpression experiments, approximately 10 pL mRNA solution (10 ng/μL) was injected into the cytoplasm of the GV oocyte. An equal amount of nuclease-free water was injected as a control.

### Western blotting

The experimental operations were based on our pre-published protocols (Gao *et al.* 2018, He *et al.* 2019). To be specific, 100 mouse oocytes were lysed in Laemmli (SDS-sample) buffer supplemented with a protease inhibitor and boiled for approximately 5 min. Samples were separated via 15% SDS-PAGE, transferred to a PVDF membrane, and blocked in 5% low-fat dry milk diluted with TBST for 1 h. Then, the samples were incubated overnight at 4°C with primary antibodies (rabbit anti-SIRT1 antibody, 1:1,000 and mouse anti-Actin antibody, 1:3,000). After three washes, secondary antibodies were applied for 1 h at 37°C, and the protein bands were detected using a Bio-Rad ChemiDoc XRS+. Membranes were washed with a stripping buffer and incubated with an anti-actin antibody as a control.

### Immunofluorescence

The procedures were conducted in accordance with our previously published protocols (Gao *et al.* 2018, He *et al.* 2019). Oocytes were fixed with 4% paraformaldehyde for 30 min, incubated with 0.5% Triton X-100 for 20 min, and 1% bovine serum albumin (diluted with phosphate-buffered saline) for 1 h. Samples were incubated with primary antibodies overnight at 4°C and with a secondary antibody for 1 h at 25°C.

For F-actin staining, MII oocytes were fixed in 3.7% paraformaldehyde for 5 min and blocked in 1% bovine serum albumin for 1 h. The samples were incubated with FITC-conjugated phalloidin for 1 h. DNA was counterstained with propidium iodide (PI) or Hoechst 33342 for 10 min. The samples were mounted on an antifade medium and examined under a laser scanning confocal microscope (Leica, Germany).

### Mitochondria distribution measurement

Mitochondria were labeled with MitoTracker. Living oocytes were cultured in M2 medium containing 200 nM MitoTracker Red (Molecular Probes, USA) for 30 min at 37°C. The DNA was counterstained with Hoechst 33342 for 10 min. The oocytes were then moved to a live-cell imaging dish after washing. Fluorescence was observed using a confocal microscope (Leica, Germany).

### Mitochondrial membrane potential measurement

Oocytes were cultured in M2 medium containing 2 μΜ JC-1 (Thermo Fisher Scientific, Cat#: T3168) for 30 min at 37°C. Chromosomes were counterstained with propidium iodide (PI) or Hoechst 33342 for 10 min. Oocytes were transferred to a live-cell imaging dish after three washes. Fluorescence was detected using a Leica inverted microscope. The fluorescence intensity was measured using ImageJ software.

### ROS measurement

Living oocytes were incubated in M2 medium with 5 μΜ CM-H_2_DCFDA (Life Technologies, Invitrogen TM, Cat#:C6827) for 30 min at 37°C in 5% CO_2_ atmosphere. Oocytes were transferred to a live-cell imaging dish after three washes and observed using a confocal microscope. The fluorescence intensity was measured using ImageJ software.

### Statistical analysis

Data were presented as mean ± SEM. Differences between two groups were analyzed by Student's *t* test. Multiple comparisons among more than two groups were analyzed by ANOVA multiple comparison test. The analyses were performed using GraphPad Prism 8 software.

## Results

### BaP disrupted mouse GV oocyte meiosis and reduced Sirt1 protein levels during IVM

**Figure 1:**
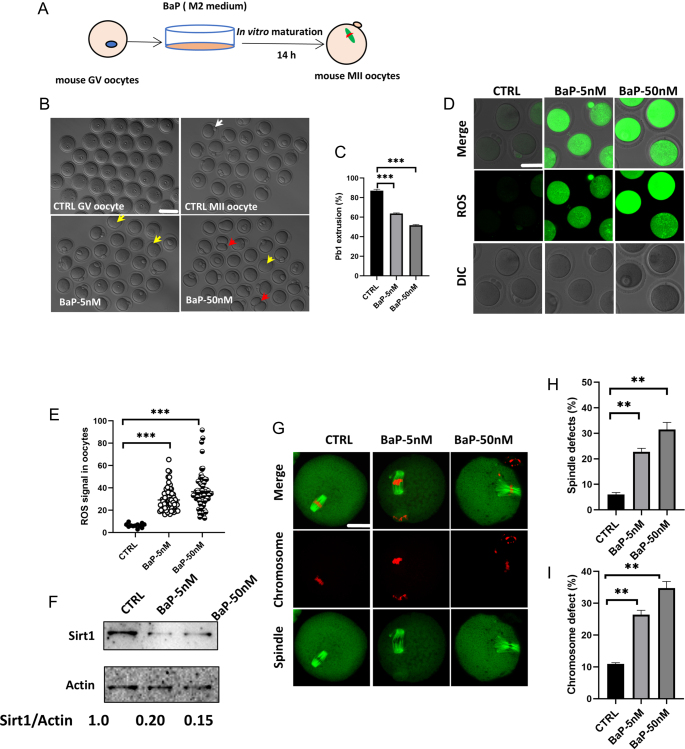
Effects of BaP exposure on mouse oocyte maturation *in vitro*. (A) A Schematic presentation of the experimental protocol in mouse oocytes. (B) Representative oocyte morphologies in different groups. Red arrowheads point to oocytes with symmetrical division or a large polar body. Yellow arrows point to oocytes with no polar body. Scale bar: 80 μm. (C) Oocyte maturation rate in the control (*n* = 199), BaP-5 nM (*n* = 176) and BaP-50 nM (*n* = 196). BaP, benzo(a)pyrene. (D) Representative MII oocytes of CM-H_2_DCFDA fluorescence. Scale bar: 80 μm. (E) Quantification of relative ROS levels in the control (*n* = 12), BaP-5 nM (*n* = 73) and BaP-50 nM (*n* = 81). Each data point represents an oocyte. (F) Sirt1 protein was verified by western blot analysis in different groups. The relative amount of SIRT1 was estimated based on the level of actin (*n* = 100 per group). (G) MII oocytes were stained with α-tubulin antibody to visualize the spindle (green) and counterstained with Hoechst 33342 (red) for chromosomes. Representative images of spindle morphologies and chromosome alignment in the control, BaP-5 nM and BaP-50 nM treated oocytes. Scale bar: 25 μm. (H) Quantification of control (*n* = 131), BaP-5 nM (*n* = 246) and BaP-50 nM (*n* = 262) with spindle disorganization. (I) Quantification of control (*n* = 137), BaP-5 nM (*n* = 192) and BaP-50 nM (*n* = 180) with chromosome defects. Data were presented as the mean percentage ± SEM of three independent experiments. *P < 0.05, **P < 0.01, ***P < 0.001.

To investigate the toxic effects of BaP exposure on mouse oocyte maturation, we utilized a concentration of 5 nM BaP and a higher concentration of 50 nM group to treat GV oocytes during IVM. As shown in Fig. 1, exposure to either 5 or 50 nM BaP prominently reduced the maturation rate (63.7 or 51.7%, respectively) compared with the control group (87%, Fig. 1A, B, C). We also found that GV oocytes exposed to BaP were predominantly arrested in the metaphase I (MI) phase after 8 h of *in vitro* culture. Approximately 34.6% oocytes exposed to BaP were arrested at the MI phase, which is significantly higher than the 4.8% observed in the control group, as shown in Supplementary Fig. 1A (see section on Supplementary materials given at the end of the article). The spindle assembly checkpoint (SAC) is a crucial protective mechanism during meiosis, primarily involved in regulating the transition from meiosis MI to anaphase I/telophase I (ATI) in oocytes. Mitotic checkpoint serine/threonine protein kinase Bub1beta (BubR1) is a key component of the SAC. Typically, the BubR1 signal disappears during MI only when the kinetochore is properly attached to microtubules. Therefore, we conducted immunofluorescence detection of BubR1 in MI oocytes to determine whether the SAC was activated. Our results showed that the fluorescence-labeled BubR1 signal was nearly undetectable on the kinetochores of control MI oocytes. In contrast, the BubR1 signal significantly increased in oocytes treated with BaP, indicating activation of the SAC system, as illustrated in Supplementary Fig. 1B and C. In summary, BaP exposure activates the SAC, leading to partial oocyte arrest during the MI phase. It is noteworthy that the SAC cannot completely prevent the transition from the MI to the ATI stage; even in the presence of errors in chromosome segregation, some oocytes are still capable of undergoing subsequent development. We will further evaluate MII-stage oocytes in our next steps.

Previous studies have found that 50 μM BaP disrupted mitochondrial distribution and function during porcine oocyte maturation (Miao *et al.* 2018). Mitochondrial dysfunction inevitably induces oxidative stress (Adhikari *et al.* 2022). Therefore, we assessed whether oxidative stress levels were elevated in oocytes exposed to low concentrations of BaP. Living oocytes were stained with CM-H_2_DCFDA to determine ROS levels. Relative quantification analysis of ROS fluorescence intensity revealed that levels were significantly elevated in oocytes exposed to 5 nM (fluorescence intensity: 29.21 ± 1.17) and 50 nM BaP (fluorescence intensity: 36.53 ± 1.65) compared to control oocytes (fluorescence intensity: 6.39 ± 0.52) (Fig. 1D and E). SIRT1 is involved in regulating ROS homeostasis, stimulating antioxidant expression, repairing cell damage and preventing cell dysfunction (Di Emidio *et al.* 2014, Singh *et al.* 2018). Di Emidio *et al.* reported that *Sirt1* is downregulated under H_2_O_2_ stress in aged mouse oocytes (Di Emidio *et al.* 2014). We found that BaP exposure significantly reduced SIRT1 protein levels in GV mouse oocytes (Fig. 1F). We further studied whether the decrease in SIRT1 destroyed the subcellular structure during the maturation of mouse oocytes. To address this issue, spindle morphology and chromosome alignment of BaP-exposed oocytes were examined. Nearly 90% of the MII oocytes in the control group had typical barrel-shaped spindles and accurate chromosome alignment (Fig. 1G). In contrast, exposure to 5 or 50 nM BaP significantly disrupted spindle formation (22.7 or 31.5%, respectively) (Fig. 1H) and increased chromosome misalignment (26.4 or 34.8%, respectively) (Fig. 1I) compared with the control. Taken together, our data indicate that BaP exposure at physiological concentrations disrupts GV oocyte maturation in mice.

### Nicotinic acid protected mouse GV oocyte IVM against BaP exposure

**Figure 2:**
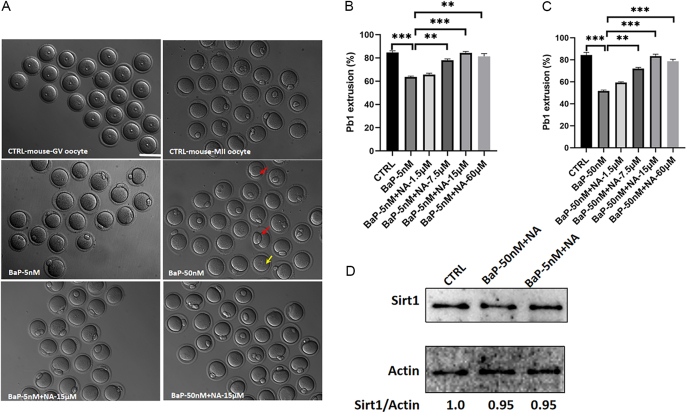
NA protected against meiotic maturation in BaP exposure mouse oocytes. (A) Representative oocyte morphologies in different groups. Red arrowheads point to oocytes with symmetrical division or a large polar body. Yellow arrows point to oocytes with no polar body. Scale bar: 80 μm. (B) NA protected against meiotic maturation in 5 nM BaP exposure mouse oocytes. The oocyte maturation rate in control (*n* = 112), BaP-5 nM (*n* = 120), BaP-5 nM + NA-7.5 μM (*n* = 191), BaP-5 nM + NA-15 μM (*n* = 173) and BaP-5 nM + NA-60 μM (*n* = 154). (C) NA protected against meiotic maturation in 50 nM BaP exposure mouse oocytes. The oocyte maturation rate in the control (*n* = 89), BaP-50 nM (*n* = 162), BaP-50 nM + NA-7.5 μM (*n* = 140), BaP-50 nM + NA-15 μM (*n* = 175) and BaP-50 nM + NA-60 μM (*n* = 160). NA, nicotinic acid. (D) Sirt1 protein was verified by western blot analysis in different groups. The relative amount of Sirt1 was estimated based on the level of actin (*n* = 100 per group). Data were presented as the mean percentage ± SEM of three independent experiments. *P < 0.05, **P < 0.01, ***P < 0.001.

NA administration can elevate NAD^+^ and Sirt1 levels in aged oocytes *in vitro* (Bertoldo *et al.* 2020, Miao *et al.* 2020, Nadeeshani *et al.* 2022). Next, we explored whether NA could alleviate meiotic arrest induced by BaP exposure. NA was diluted to a series of concentrations (7.5, 15 and 60 μM) in a culture medium containing either 5 or 50 nM BaP (Fig. 2A and Supplementary Fig. 2). Our data indicate that the maturation rate of oocytes exposed to BaP increased with NA supplementation. As shown in Fig. 2B and C, a concentration of 15 μM NA significantly enhanced the extrusion of the first polar body in both the 5 nM BaP (84.3%) and 50 nM BaP exposure groups (83.3%) when compared to other concentrations. Moreover, 60 μM NA did not obviously increase the oocyte maturation ratio in the 5 nM (81.3%) and 50 nM BaP exposure groups (78.7%) compared with 15 μM NA. Treatment with 15 and 60 μM NA yielded similar results in the 5 and 50 nM BaP exposure groups. Therefore, a treatment of 15 μM NA was selected for further experiments. We observed that 15 μM NA significantly increased Sirt1 expression in both GV and MII oocytes, with expression levels comparable to those in the control group (Fig. 2D and Supplementary Fig. 3). In addition, lower ROS levels were observed in the 5 nM BaP + NA group (7.31 ± 0.34) or the 50 nM BaP + NA group (7.179 ± 0.3481), as detected by fluorescence intensity quantification (Supplementary Fig. 4). To clarify whether NA supplementation reduces ROS by increasing Sirt1 levels, we intended to inject *Sirt1* mRNA in BaP-exposed GV oocytes and detected ROS levels. We conducted a western blot analysis to confirm the successful overexpression of Sirt1 using the Sirt1 antibody in Supplementary Fig. 5A. Our results show that Sirt1 overexpression could partly reduce the BaP-induced ROS in Supplementary Fig. 5B and C. The mitochondrial membrane potential serves as a crucial indicator of mitochondrial function. To determine mitochondrial membrane potential, the oocytes were labeled with the fluorescent dye JC-1. JC-1 monomers exhibit increased green fluorescence signals, while JC-1 aggregates, which accumulate in the mitochondrial matrix and display red fluorescence signals. The JC-1 red/green fluorescence ratio represents the membrane potential state. Mouse oocytes exposed to 5 nM BaP showed lower mitochondrial membrane potential (JC-1 red/green fluorescent ratio 0.57), while the control group had a JC-1 red/green fluorescent ratio was 1.45 (Supplementary Fig. 6). NA supplementation could partly rescue the lower mitochondrial membrane potential in mouse oocytes (JC-1 red/green fluorescent ratio: 1.31). Taken together, our data suggest that NA can at least partially rescue the maturation of mouse GV oocytes treated with BaP.

### Nicotinic acid rescued mitochondrial dysfunction and DNA damage in BaP-treated mice during GV oocyte maturation

**Figure 3:**
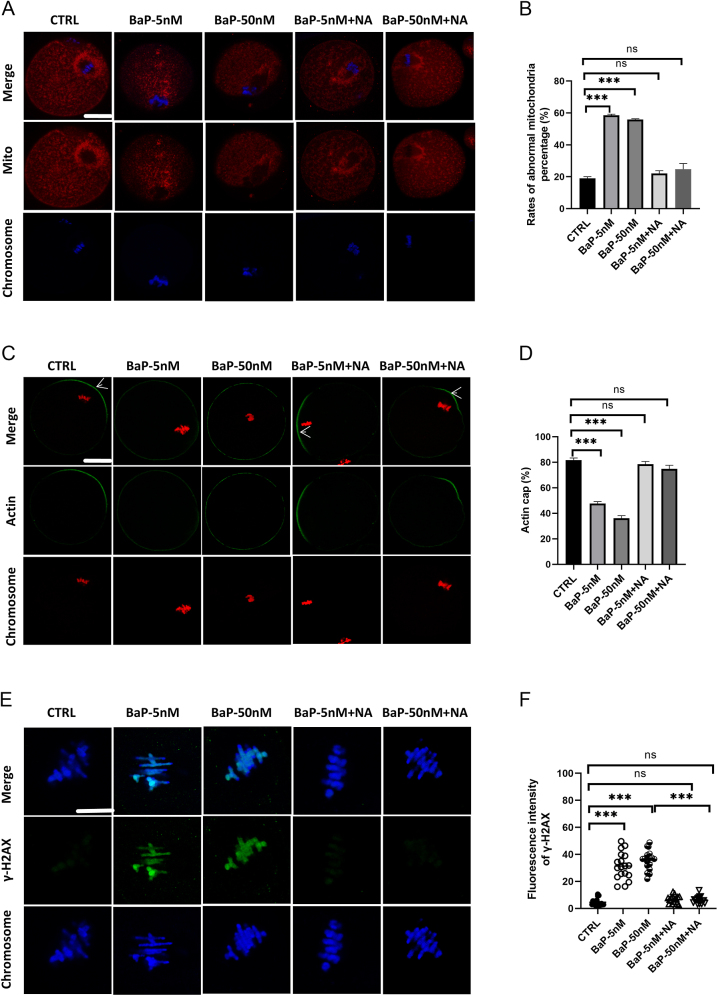
Effects of NA on the subcellular structure in BaP-exposed mouse oocytes. (A) MII oocytes were labeled with MitoTracker Red to visualize mitochondrial distribution and counterstained with Hoechst 33342 (blue) for chromosomes. Representative images of mitochondrial distribution patterns in MII oocytes. Scale bar: 25 μm. (B) Quantification of control (*n* = 180), BaP-5 nM (*n* = 125), BaP-50 nM (*n* = 101), BaP-5 nM + NA (*n* = 81) and BaP-50 nM + NA (*n* = 84) with each mitochondrial distribution pattern. (C) MII oocytes were labeled with phalloidin to visualize actin (green) and were counterstained with propidium iodide (PI) (red) for chromosomes. White arrowheads point to MII oocytes with actin filaments on the membrane. Scale bar: 25 μm. (D) Quantitative analysis of control (*n* = 115), BaP-5 nM (*n* = 120), BaP-50 nM (*n* = 126), BaP-5 nM + NA (*n* = 124) and BaP-50 nM + NA (*n* = 141) with normal actin cap formation. (E) MII oocytes were stained with anti-γ-H2A.X antibody to visualize DNA damage (green) and counterstained with Hoechst 33342 (blue) for chromosomes. Scale bar: 50 μm. (F) Quantification of the fluorescence intensity of DNA damage in control (*n* = 17), BaP-5 nM (*n* = 18), BaP-50 nM (*n* = 17), BaP-5 nM + NA (*n* = 17) and BaP-50 nM + NA (*n* = 17). Data were presented as mean ± SEM of three independent experiments. *P < 0.05, **P < 0.01, ***P < 0.001.

NA can effectively reverse BaP-induced oxidative stress and increase SIRT1 levels in mouse oocytes. Next, we examined whether NA could ameliorate subcellular structural abnormalities and DNA damage. MII oocytes were labeled with MitoTracker to assess mitochondrial dynamics (Fig. 3A). Almost 80% of the MII oocytes in the control group showed typical accumulation of mitochondria at the spindle periphery. In contrast, we observed approximately 58.5 and 62.9% abnormal mitochondrial distribution in oocytes exposed to 5 and 50 nM BaP, respectively (Fig. 3B). However, we found that ∼22% of the abnormal distribution pattern was markedly decreased in the 5 nM BaP + NA and 50 nM BaP + NA groups compared with the BaP exposure. These results suggest that IVM supplemented with NA improves mitochondrial distribution and function. Next, we explored whether NA supplementation could reduce abnormalities in oocyte meiosis. Mitochondria provide energy for oocyte cytoskeletal organization. The markedly decreased abnormalities in spindle assembly and chromosome defects caused by 5 and 50 nM BaP are shown in Supplementary Fig. 7A. In particular, only 7.7 and 9.2% abnormal spindles were detected in the 5 nM BaP + NA and 50 nM BaP + NA groups, respectively (Supplementary Fig. 7B). Similarly, a significant decrease in chromosome misalignment was observed in both groups treated with NA (13.7 or 13.2%) (Supplementary Fig. 7C).

Next, we investigated whether BaP exposure caused first polar body (Pb1) extrusion disorder caused by actin dynamics. F-actin was labeled with phalloidin (Fig. 3C). We observed that the weak signals of actin filaments on the membrane were present in the 5 nM BaP-treated oocytes (47.7%) and the 50 nM BaP-treated group (36%), compared to the control group (81.7%) (Fig. 3D). Notably, NA supplementation remarkably increased the intensity of actin filaments signals in the 5 nM (78.5%) and 50 nM BaP treatment groups (74.8%) compared with the control group. These data suggest that NA restored spindle assembly, chromosome alignment and reversed actin filament defects induced by BaP exposure during mouse oocyte maturation.

High levels of oxidative stress can also induce DNA damage. Phosphorylation of the Ser-139 residue of the histone variant H2AX forms γ-H2A.X, which is a marker for monitoring DNA damage initiation and resolution. Thus, we examined whether exposure to 5 and 50 nM BaP induced DNA damage in MII oocytes (Fig. 3E). Our data demonstrated that γ-H2A.X signals were significantly increased following treatment of oocytes with 5 nM (fluorescence intensity: 31.36 ± 2.34) and 50 nM (fluorescence intensity: 35.72 ± 1.81) BaP relative to that of the control (fluorescence intensity: 4.65 ± 0.66) (Fig. 3F). However, NA protected GV oocytes from DNA damage induced by exposure to 5 nM BaP (fluorescence intensity: 6.57 ± 0.71) and 50 nM BaP (fluorescence intensity: 6.34 ± 0.62), significantly reducing γ-H2A.X signals. Our results indicate that NA partly alleviated subcellular structural abnormalities and DNA damage in BaP-exposed mouse oocytes.

### Nicotinic acid protected human GV vesicle oocyte meiosis against toxicity of BaP during maturation

**Figure 4:**
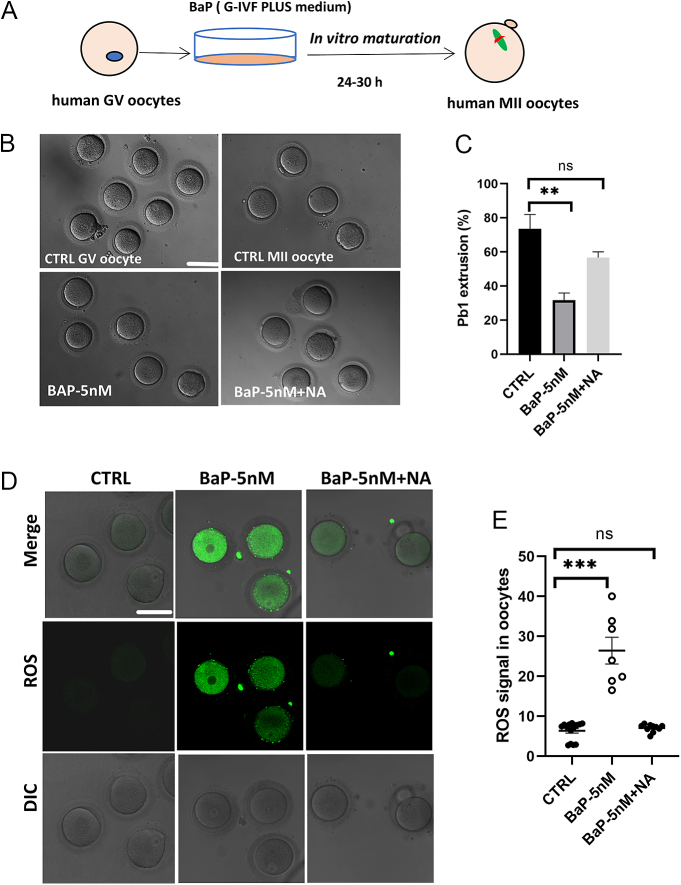
NA protected against meiotic maturation in BaP exposure human oocytes. (A) Schematic presentation of the experimental protocol in human oocytes. (B) Representative oocyte morphologies in different groups. Scale bar: 100 μm. (C) The oocyte maturation rate in control (*n* = 21), BaP-5 nM (*n* = 22) and BaP-5 nM + NA (*n* = 16). (D) Representative images of CM-H_2_DCFDA fluorescence. The samples were GV oocytes cultured *in vitro* for 24–30 h. Scale bar: 100 μm. (E) The fluorescence intensity of ROS in control (*n* = 15), BaP-5 nM (*n* = 7) and BaP-5 nM + NA (*n* = 10). Each data point represents an oocyte. Each data point represents an oocyte. Data were presented as mean ± SEM of three independent experiments. *P < 0.05, **P < 0.01, ***P < 0.001.

Women exposed to mainstream smoke have approximately 5 nM BaP in their follicular fluid (Neal *et al.* 2008). Thus, we explored the toxic effects of 5 nM BaP on human GV oocyte maturation (Fig. 4A). Clinically discarded GV oocytes from intra-cytoplasmic sperm injection/IVF were used. Approximately 73.5% GV oocytes extruded Pb1 in the control group; however, 5 nM BaP exposure significantly decreased the maturation rate of human GV oocytes (31.7%) (Fig. 4B and C). Since 15 μM NA could alleviate the impaired mouse oocyte quality caused by 5 and 50 nM BaP, we treated human GV oocytes exposed to 5 nM BaP during IVM with 15 μM NA. NA improved Pb1 extrusion in BaP-exposed oocytes (56.7%) compared with that in the BaP-exposed oocytes (Fig. 4C). After 24–30 h of IVM, MII oocytes were stained with the fluorescent dye CM-H_2_DCFDA to determine ROS levels. Consistent with the phenotype of mouse oocytes, treatment of human GV oocytes with 5 nM BaP induced a significant increase in ROS levels (fluorescence intensity: 26.43 ± 3.36) compared to that in the control (fluorescence intensity: 6.367 ± 0.5762) (Fig. 4D and E). Overall, these results suggest that BaP exposure inhibited human GV oocyte maturation, and we found that 15 μM NA administration could also rescue it. As expected, NA supplementation significantly decreased ROS levels (fluorescence intensity: 7.04 ± 0.30, Fig. 4D and E). Mitochondrial membrane potential is an important marker of mitochondrial function. To determine mitochondrial membrane potential, oocytes were labeled with the fluorescent dye JC-1. JC-1 monomers showed more green fluorescence signals, whereas JC-1 aggregates gathered in the mitochondrial matrix showed red fluorescence signals. The JC-1 red/green fluorescence ratio represents membrane potential. Human oocytes exposed to 5 nM BaP showed lower mitochondrial membrane potential, as indicated by green fluorescence (JC-1 red/green fluorescence ratio of 0.63), while the control group showed red fluorescence (JC-1 red/green fluorescence ratio of 1.51) (Supplementary Fig. 8A and B). We found that NA supplementation could partially restore mitochondrial membrane potential in several oocytes; however, some of these oocytes remained abnormal (Supplementary Fig. 8A and B). This discrepancy may be attributed to variations in the quality of the human GV collected clinically. Collectively, these data suggest that NA supplementation partially enhances the quality of BaP-exposed human GV oocytes during IVM.

## Discussion

Oocyte competence is determined by multiple factors in the follicular environment, for example, hormones, growth factors and metabolites (Ahmed *et al.* 2020). Here, we aimed to demonstrate that exposure to follicular fluid concentrations of BaP decreased the maturation of GV oocytes in both mice and humans. This effect was associated with increased ROS levels and disruption of mitochondrial functions and spindle assembly. Importantly, supplementation with NA enhanced Sirt1 expression and mitigated the toxicity of BaP during IVM.

Smoke produced by tobacco can be divided into mainstream smoke and sidestream smoke. Secondhand smoke is formed through the combination of mainstream smoke exhaled by active smokers (initially inhaled from the cigarette filter tip) and sidestream smoke emitted from the smoldering cigarette, which subsequently mixes with ambient air. Gas chromatography-mass spectrometry was employed to quantify the levels of benzo(a)pyrene (BaP) in the follicular fluid of women undergoing IVF, specifically comparing those exposed to mainstream smoke (*n* = 19) with non-smokers (*n* = 10). The concentration of BaP in the follicular fluid of women who smoke was approximately 5 nM (Tuttle *et al.* 2009). To better simulate the physiological environment of GV oocyte exposure in female smokers, we utilized 5 nM BaP and a relatively high concentration of 50 nM as a control in mouse GV oocytes. We then investigated the effect of 5 nM BaP on human GV oocyte maturation. Our results revealed similarities between mouse oocytes exposed to 5 and 50 nM BaP and human oocytes exposed to 5 nM BaP, including a decreased maturation rate and increased oxidative stress. Therefore, mouse GV oocytes serve as a suitable model for toxicity studies in this context.

NAD^+^ is one of the key molecules that controls mitochondrial oxidative metabolism during oocyte development (Miranda-Vizuete *et al.* 2000). When released into the extracellular environment, it is also a substrate for a variety of signaling enzymes and regulates multiple cellular signaling pathways, including deacetylases (sirtuins), ADP-ribosyl transferases and circulating ADP-ribose synthetases (Yang & Sauve 2016, Johnson & Imai 2018, Hamaidi & Kim 2022). Of these, SIRT1 is the NAD^+^-dependent deacetylase, which is responsible for alleviating oxidative stress in ovarian cells through deacetylation of members of the forkhead box transcription factor (FOXO) family (Hori *et al.* 2013, Iyer *et al.* 2014, Nishigaki *et al.* 2022). Sirt1 serves as a crucial defense factor against ROS through the FoxO3a-MnSOD axis during the maturation of mouse GV oocytes (Lin *et al.* 2014). SIRT1 plays a significant role in maintaining ROS homeostasis by stimulating antioxidant expression, repairing cellular damage and preventing cellular dysfunction (Di Emidio *et al.* 2014, Singh *et al.* 2018). Furthermore, reduced SIRT1 levels contribute to mitochondrial dysfunction by elevating ROS production, lipid peroxidation and DNA damage in oocytes, ultimately leading to infertility (Alam *et al.* 2021). Several antioxidant supplements that improve mitochondrial function *in vitro* have been shown to increase the developmental competence of oocytes or embryos (Xu *et al.* 2018). Consistent with this notion, we found that *Sirt1* expression decreased following treatment of mouse oocytes with 5 nM BaP. We also use the expression level of SIRT1 protein as one of the indicators for evaluating the quality of oocytes. It has been shown that the expression of SIRT1 is reduced, leading to spindle defects, chromosome misalignment and disturbed redistribution of cortical granules and mitochondria in vitro-aged porcine oocytes (Ma *et al.* 2015). Our results indicate that the concentration of BaP in follicular fluid adversely affect subcellular structures and cause DNA damage during mouse oocyte maturation.

SIRT1 serves as a universal mediator that exerts metabolic effects in a tissue-dependent manner in response to changes in the systemic levels of NAD^+^ (Imai 2009). Therefore, NAD^+^ supplementation can be used to decrease oocyte ROS levels. Nicotinic acid administration during *in vitro* culture improves oocyte quality by elevating NAD^+^ and SIRT1 levels in aged oocytes (Wu *et al.* 2019). Collins *et al.* reported that NA is a more favorable precursor than nicotinamide (NAM) in the liver, intestine and kidneys (Collins & Chaykin 1972). NA supplementation increases the bioavailability of NAD^+^ precursors in the follicular fluid of the dominant follicle, which contributes to improved oocyte quality, especially in older oocytes (Pollard *et al.* 2022). In addition, NA treatment had a more favorable action in correcting meiosis in high-fat diet (HFD) mouse oocytes compared to nicotinamide mononucleotide (NMN) (Wang *et al.* 2021). Given that NA is more effective when supplemented *in vitro*, we opted to use NA to supplement NAD^+^ and assessed its effects by measuring SIRT1 protein levels. Our data also showed that NA supplementation restored SIRT1 levels, lowered ROS production and ameliorated meiotic defects in BaP-exposed mouse oocytes. Importantly, NA not only enhanced human GV oocyte maturation but also protected against mitochondrial dysfunction and oxidative stress in BaP-treated GV oocytes.

For future studies, it is essential to obtain human GV oocytes of homogeneous quality in clinical settings, which necessitates additional ethical approval. This study aims to evaluate the effects of BaP exposure on embryo developmental potential and the influence of NA treatment. Our findings offer new insights into enhancing clinical outcomes of human GV oocyte IVM in assisted reproduction. Furthermore, the study suggests that NA supplementation improves oocyte quality in patients who are either active or passive smokers, thereby enhancing reproductive outcomes in these populations.

## Conclusion

**Figure 5:**
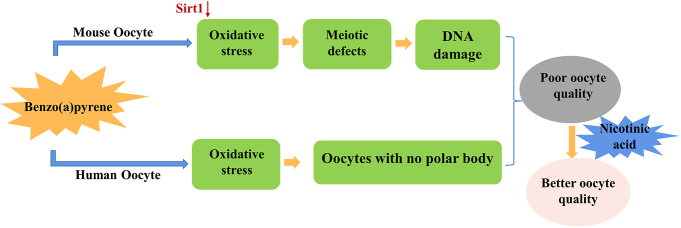
Diagram of the experiment in mouse and human oocytes.

In summary, the concentrations of BaP in follicular fluid from smoking-exposed populations induce meiotic defects by increasing oxidative stress in both mice and humans. NA partially mitigates BaP toxicity during IVM by protecting GV oocyte meiosis (Fig. 5).

## Supplementary materials



## Declaration of interest

The authors declare that there is no conflict of interest that could be perceived as prejudicing the impartiality of the work reported.

## Funding

This research was funded by the Guangdong Basic and Applied Basic Research Foundationhttps://doi.org/10.13039/501100021171 (2022A1515140059 and 2023A1515111005), the National Natural Science Foundation of Chinahttps://doi.org/10.13039/100017052 (82271688), the key project of Huizhou Science and Technology R&D Program (2022CZ010001), the Huizhou Central People's Hospital Dengfeng Project of Guangdong (2019–2022) and the Joint Program on Health Science & Technology Innovation of Hainan Province (WSJK2024QN027).

## Author contribution statement

JH and ZC conceived and designed the project. MG, DL, YQ, SZ, BC and TC performed the experiments. MG, DL, SZ, YQ, BC and TC analyzed the data. JH and MG wrote the manuscript. JH and ZC contributed to the final approval of the manuscript. All authors have read and agreed to the published version of the manuscript.

## Data availability

All data generated or analyzed during this study are available from the corresponding author on reasonable request.
