# Supplementary material for: Nicotinic acid protects germinal vesicle oocyte meiosis against toxicity of benzo(a)pyrene in mice and humans
Source: Reproduction. 2025 Mar 19;169(4):e240364. doi: 10.1530/REP-24-0364 (PMC11949519; doi:10.1530/REP-24-0364)
Supplement: Supplementary file 1 [file supplementary_materials.pdf]

Supplementary Material

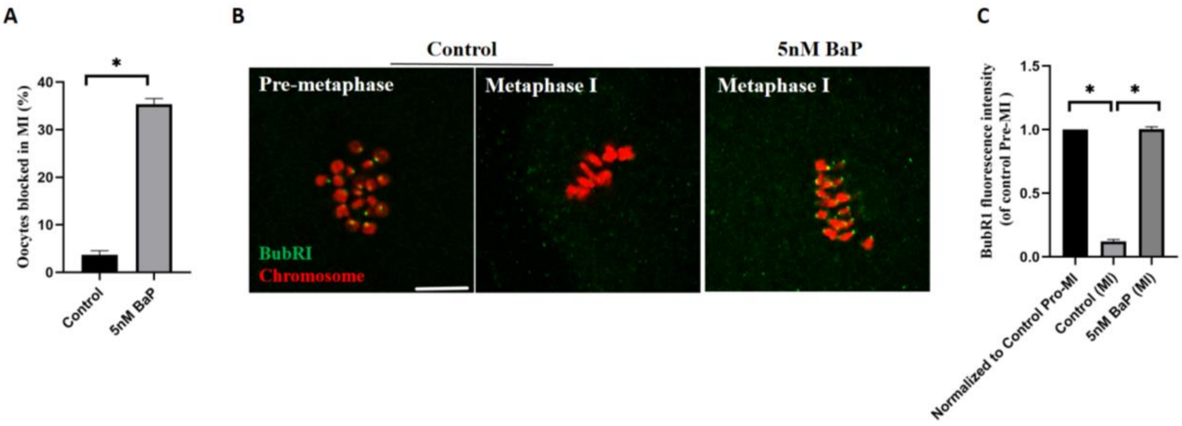

Supplementary Figure 1. BaP exposed activates the SAC in mouse oocytes. (A) The percentage of oocytes arrested at metaphase I in control and BaP exposed oocytes. (B) Control and BaP exposed oocytes were stained with anti-BubR1 antibody (green) and counterstained with propidium iodide to visualize the chromosomes (red). Representative images of pre-metaphase I and metaphase I oocytes are shown. (**C**) Quantitative analysis of BubR1 fluorescence intensity in control (n=16) and BaP exposed (n=28) oocytes. Data are expressed as the mean±SEM of three independent experiments. *, $p<0.05$. Scale bar: 5μm.

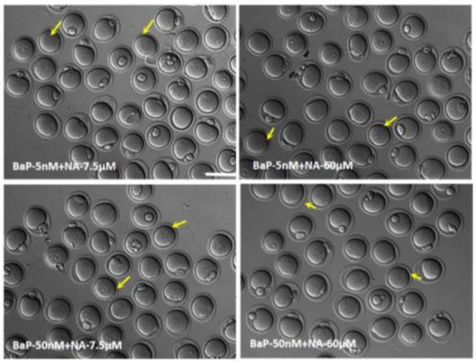

Supplementary Figure 2. NA protected against meiotic maturation in BaP exposure mouse oocytes. (**A**) Representative oocytes morphologies in the BaP-5nM+NA-7.5μM (n=191), BaP-5nM+NA-60μM (n=154), BaP-50nM+NA-7.5μM (n=140), BaP-50nM+NA-60μM (n=160). Yellow arrows point to oocytes with no

polar body. Scale bar :80 µm.

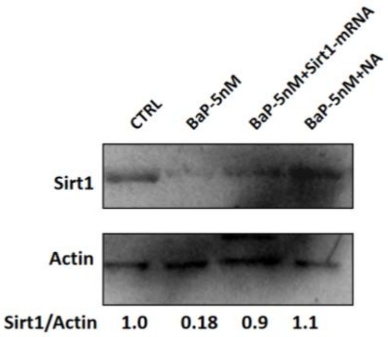

Supplementary Figure 3. Nicotinic acid rescued poor quality of MII oocytes induced by BaP exposure. Sirt1 protein was verified by western blot analysis in different groups. The relative amount of Sirt1 was estimated based on the level of actin (n = 70 per group). Data were showed as mean ± SEM of three independent experiments. *p < 0.05. **p < 0.01. ***p < 0.001.

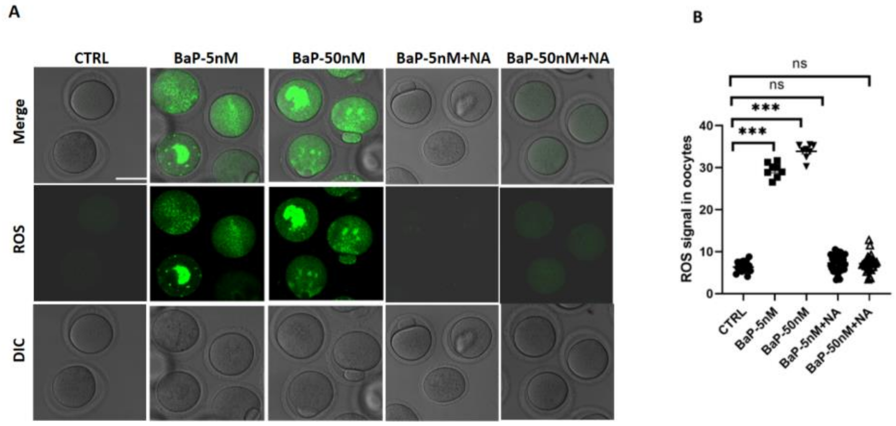

Supplementary Figure 4. Effects of NA on the level of ROS in BaP-exposed mouse MII oocytes. (**A**) Representative images of CM-H_2DCFDA fluorescence. Scale bar: 50 µm. (**B**) Quantification of the relative levels of ROS in control (n=16), BaP-5nM (n=8), BaP-50nM (n=8), BaP-5nM+NA (n=33), BaP-50nM+NA (n=31). Each data point represents an oocyte. Scale bar: 80 µm. Data were showed as mean ± SEM of three independent experiments.

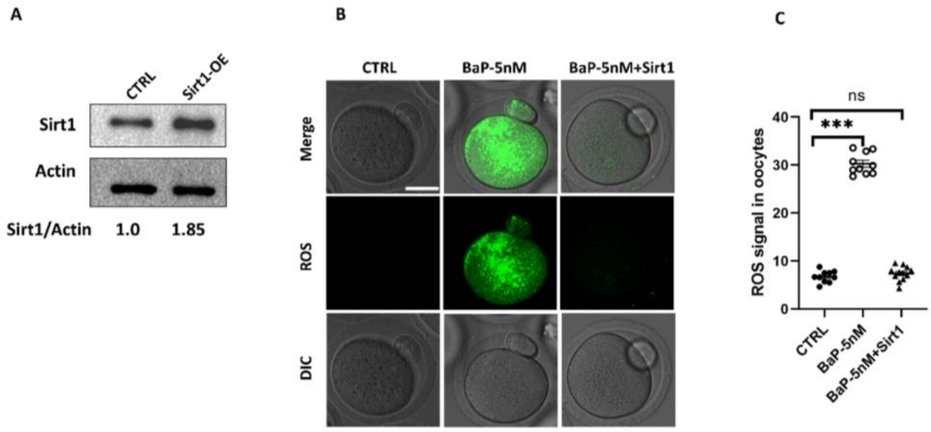

Supplementary Figure 5. Effects of Sirt1 overexpression on the level of ROS in BaP-exposed mouse oocytes. (**A**) Western blot analysis confirming successful overexpression of Sirt1 using with an anti- Sirt1 antibody (n = 80 per group). (**B**) Representative images of CM-H_2DCFDA fluorescence in MII oocytes. Scale bar: 25 μm. (**C**) Quantification of the relative levels of ROS in control (n=10), BaP-5nM (n=10), BaP-50M+Sirt1 (n=12). Each data point represents an oocyte. Data were showed as mean ± SEM of three independent experiments.

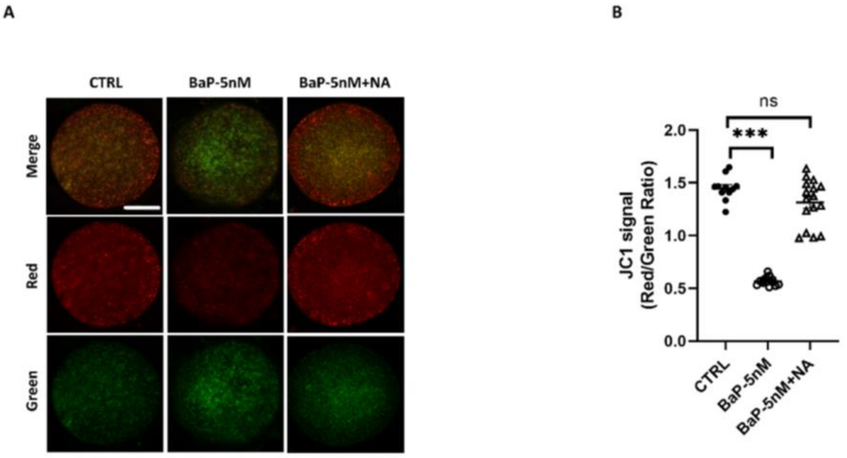

Supplementary Figure 6. NA protected against lower mitochondrial membrane potential in BaP exposure mouse oocytes. (**A**) Mitochondrial membrane potential measured by JC-1 fluorescence in MII oocytes. The green fluorescence were inactive mitochondria and the red fluorescence were active mitochondria in oocytes. Scale bar :25 μm. (**B**) Histogram showing the JC-1 red/green fluorescence ratio, control (n=11), BaP-5nM (n=13), BaP-5nM+NA (n=16). Each data point represents an oocyte. Data were showed as mean ± SEM of three independent experiments. *$p < 0.05$. **$p < 0.01$. ***$p < 0.001$.

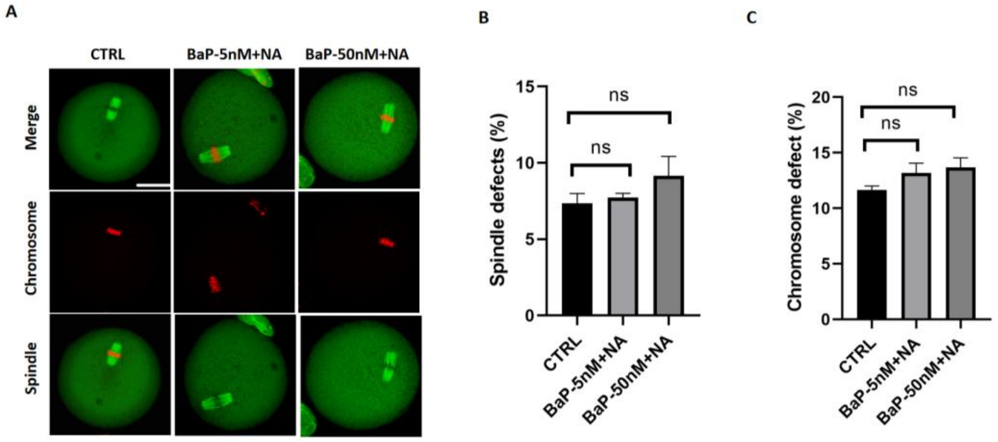

Supplementary Figure 7. Effects of NA on the cytoskeletal organization in BaP-exposed mouse MII oocytes. (**A**) Representative images of spindle morphologies (green) and chromosome alignment (red). Scale bar:25μm. (**B**) Quantification of control (n=95), BaP-5nM+NA (n=192), BaP-50nM+NA (n=210) with spindle disorganization. (**C**) Quantification of control (n=94), BaP-5nM+NA (n=190), BaP-50nM+NA (n=158) with chromosome defects. Data were showed as mean percentage ± SEM of three independent experiments.

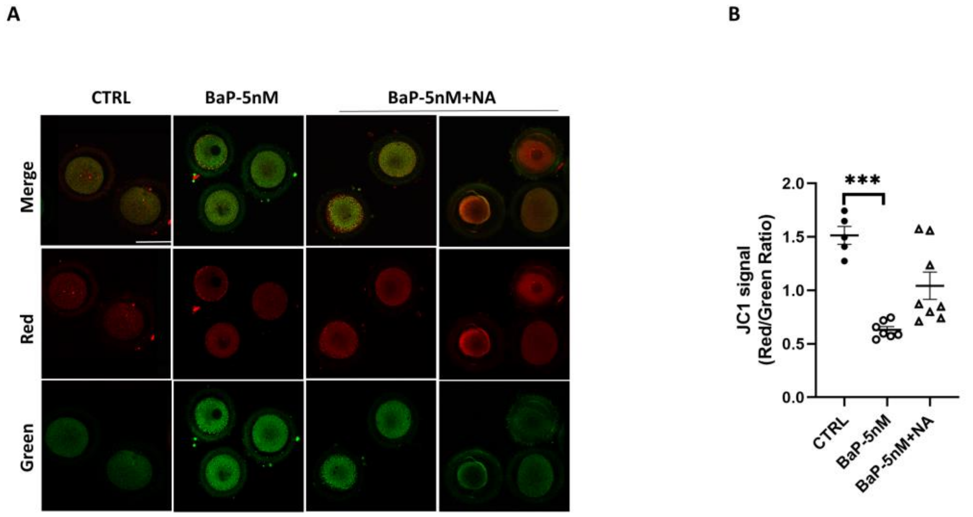

Supplementary Figure 8. NA protected against meiotic maturation in BaP exposure human MII oocytes. (**A**) Mitochondrial membrane potential measured by JC-1 fluorescence. The green fluorescence were inactive mitochondria and the red fluorescence were active mitochondria in oocytes. Scale bar :100 μm. (**B**) Histogram showing the JC-1 red/green fluorescence ratio, control (n=5), BaP-5nM (n=7), BaP-5nM+NA (n=8). Each data point represents an oocyte. Data were showed as mean ± SEM of three independent experiments. *p < 0.05. **p < 0.01. ***p < 0.001.

Original western blots

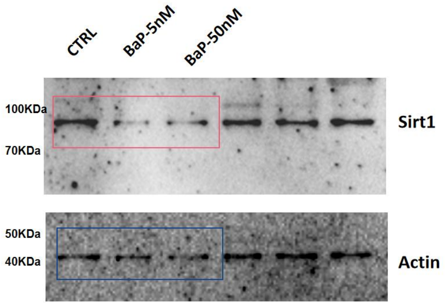

Original western blots about corresponding to Fig1 F in the manuscript.

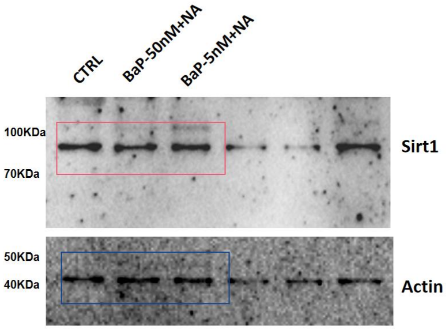

Original western blots about corresponding to Fig2 D in the manuscript.

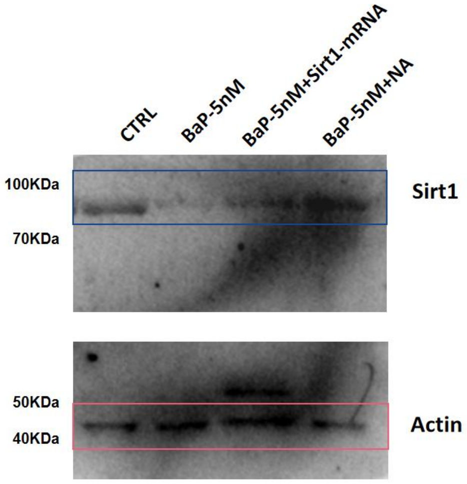

Original western blots about corresponding to Supplementary Figure 3A in the manuscript.
